# Determinants of old age disability in Botswana: an empirical investigation using generalized linear models

**DOI:** 10.1186/s12877-025-06534-z

**Published:** 2025-11-07

**Authors:** Tiro Theodore Monamo, Mpho Keetile, Gobopamang Letamo

**Affiliations:** Department of Population Studies, University of Botswana, Gaborone, Private Bag, 00705, Botswana

## Abstract

**Background:**

As Botswana experiences a demographic transition marked by an expanding population of older adults, understanding the determinants of disability among older adults becomes critical for shaping inclusive health and social policies. Disability in later life often stems not only from biological ageing but also from intersecting personal, household, and community-level conditions. Despite increasing attention to ageing in sub-Saharan Africa, few studies have comprehensively assessed the multilevel factors influencing functional limitations in older populations.

**Methods:**

Drawing on nationally representative microdata from the 2022 Botswana Population and Housing Census, this study employed Generalized Linear Models (specifically Poisson regression) to examine the severity of disability among individuals aged 65 years and above. Disability was measured using a composite count variable derived from three functional domains: mobility, self-care, and cognition. The final analytical sample comprised 47,309 older adults. The model integrated a wide range of covariates across individual, household, and community levels. Model selection was based on goodness-of-fit statistics, including AIC, BIC, and deviance diagnostics.

**Results:**

The multilevel Poisson model revealed that age, gender, education, marital status, and employment status were significant individual-level predictors of disability. Older adults aged 65–69 and 70–74 were significantly less likely to experience multiple disabilities compared to those aged 80+, with IRRs of 0.434 and 0.576 respectively. Males had lower disability counts than females (IRR = 0.648), and those with only primary or less education had higher disability rates than those with tertiary education (IRR = 1.207). At the household level, individuals in smaller households (number of rooms) reported significantly higher disability levels. At the community level, urban residence, access to electricity, internet, and transportation services were all associated with reduced disability. Although interaction terms were not explicitly specified, the integrated model structure supported inferences about cross-level interactions between environmental infrastructure and individual vulnerabilities.

**Conclusion:**

This study provides a comprehensive analysis of the multifactorial determinants of old-age disability in Botswana. The findings underscore the need for integrated, multisectoral strategies that go beyond healthcare access to include educational equity, age-friendly infrastructure, digital inclusion, and gender-sensitive social protection. Policies must address not only individual risk but also household and community conditions that jointly shape disability outcomes. These insights provide a critical roadmap for building age-inclusive societies as Botswana continues to age.

**Supplementary Information:**

The online version contains supplementary material available at 10.1186/s12877-025-06534-z.

## Introduction

Ageing is a universal and inevitable process that brings about significant transformations in physical functioning, cognitive abilities, and social integration. One of the most profound challenges associated with ageing is the onset of disability, which adversely affects independence, quality of life, and overall well-being among older adults [1]. As populations age globally, including in low- and middle-income countries like Botswana, understanding the prevalence, distribution, and determinants of disability among older persons is crucial for informing inclusive, evidence-based policies and interventions.

Botswana is currently undergoing a demographic transition characterized by an increasing proportion of older adults [2]. According to the 2022 Botswana Population and Housing Census, individuals aged 65 years and above comprise approximately 5.5% of the total population, a figure expected to grow significantly in the coming decades. This demographic shift is accompanied by rising rates of age-related health conditions [3], presenting new and complex challenges for individuals, families, healthcare systems, and policymakers. Estimates from the UN DESA and Population Division [4] indicate that more than 46% of adults aged 60 years and above live with a disability globally, with prevalence rates even higher in sub-Saharan Africa due to limited access to health care, preventive services, and assistive technologies.

Disability in later life can result from multiple causes, including chronic non-communicable diseases, injuries, cumulative occupational exposures, and the natural processes of biological ageing [5]. Among older adults in Botswana, common functional limitations include mobility impairments, visual and auditory deficits, and cognitive decline [6]. These impairments compromise the ability to perform activities of daily living (ADLs), reduce social participation, and increase reliance on family caregivers and formal support systems.

The sociocultural landscape of Botswana plays a central role in shaping the lived experiences of older adults with disabilities. Traditionally, older persons were cared for within extended family structures, with respect and caregiving embedded in Setswana norms and communal values [7]. However, trends in urbanization, labour migration, and modernization have contributed to the weakening of traditional support systems, resulting in growing numbers of older adults who must navigate disability and ageing with diminished familial and institutional support [8]. This evolving context underscores the urgency of strengthening health systems, expanding social protection measures, and developing community-based interventions that prioritize the dignity and autonomy of older adults.

Theoretically, this study is grounded in the World Health Organization’s International Classification of Functioning, Disability and Health (ICF) model, which provides a comprehensive framework for understanding disability as the result of interactions between health conditions, personal factors, and environmental influences [9]. The ICF model is further enriched by integrating Age Stratification Theory and the Person-Environment Fit Theory. Age Stratification Theory emphasizes how ageing and disability experiences differ across generational cohorts due to varying historical and social conditions [10]. The Person-Environment Fit Theory highlights the importance of the equivalence between an individual’s capacities and their physical and social environments [11], reinforcing the ICF’s ecological approach. Together, these frameworks offer a multidimensional lens to assess how structural, societal, and individual-level factors intersect to produce disability outcomes in later life.

Alongside this framework, this study posits that the combination of individual, household, and community-level factors provides a more comprehensive and accurate prediction of disability outcomes among older adults in Botswana than any single level of analysis alone. This hypothesis underpins the analytical approach and reflects the complexity of disability as a biopsychosocial phenomenon influenced by intersecting determinants across multiple contexts. Therefore, the study aims to examine the determinants of disability among older adults in Botswana, with a particular focus on individual, household, and community-level influences. Using micro data from the 2022 Botswana Census, the study employs advanced statistical modelling, specifically generalized linear models, to estimate the prevalence and severity of disability.

By synthesizing demographic and public health perspectives with sophisticated modelling approaches, this research contributes to the growing body of literature on ageing and functional health in sub-Saharan Africa. The findings are intended to inform policy actors, service providers, and community-based stakeholders about the multifaceted nature of disability among Botswana’s ageing population. Ultimately, the study seeks to foster a more inclusive and supportive social environment for older persons with disabilities, ensuring their needs are prioritized in national development agendas.

### Theoretical framework

Understanding the determinants of disability among older adults requires a multidimensional theoretical lens that captures the interplay between individual characteristics, environmental influences, and broader societal structures. This study is grounded in three complementary theoretical models: the International Classification of Functioning, Disability, and Health (ICF) Framework, the Age Stratification Theory of Ageing, and the Person–Environment Fit Theory of Ageing.

Together, these frameworks offer an integrated perspective on how functional limitations emerge and evolve over time, shaped by health conditions, sociohistorical contexts, and the alignment between personal capacities and environmental demands. The application of these models enables a comprehensive analysis of disability as not merely a clinical outcome, but as a dynamic, socially embedded process influenced by multilevel determinants. The subsections below outline each theoretical model and its relevance to the study of ageing and disability in Botswana.

### International classification of functioning, disability, and health (ICF) framework

The International Classification of Functioning, Disability, and Health (ICF), developed by the World Health Organization (WHO) in 2001, provides a comprehensive and dynamic framework for understanding human functioning and disability. Rather than viewing disability solely as a consequence of disease or impairment, the ICF conceptualizes it as a multidimensional phenomenon resulting from the complex interaction between an individual’s health condition and a wide array of contextual factors. This holistic perspective acknowledges that disability is not inherent to the individual alone but is shaped by the broader environment in which they live and operate.

According to the ICF, contextual factors are branched into environmental and personal components. Environmental factors encompass all external influences (physical, social, and attitudinal) that affect how individuals experience and interact with the world. These include immediate settings such as the home, workplace, and school, as well as broader societal elements like healthcare systems, public infrastructure, social norms, and legislative frameworks. The ICF distinguishes environmental influences at two levels: the individual level, referring to the person’s immediate surroundings, and the societal level, encompassing the wider socio-political and cultural context. These environmental factors may function either as facilitators that support participation and independence, or as barriers that hinder activity and inclusion.

The ICF also emphasizes the role of personal factors, which include attributes such as age, gender, education, coping styles, and personality traits. While these are not classified within the ICF classification due to their variability across cultures and contexts, they are recognized as essential elements that interact with health and environmental conditions to influence an individual’s overall functioning.

Importantly, the ICF reframes disability as the outcome of mismatches between a person’s capacities and the demands or constraints of their environment. For instance, two individuals with the same medical condition may experience vastly different levels of disability depending on the accessibility of their physical surroundings, the availability of assistive technologies, the supportiveness of social structures, and their own personal resources. Thus, the ICF promotes an integrated biopsychosocial model that guides both assessment and intervention strategies by encouraging a shift from solely treating impairments to modifying environments and enhancing individual resilience and participation.

### Age stratification theory of ageing

The Age Stratification Theory of Ageing, developed in the early 1970 s, offers a sociological perspective on the interplay between ageing individuals and the broader societal structures in which they are embedded. This theory marked a significant shift from solely biological or psychological explanations of ageing by emphasizing the dynamic, reciprocal relationship between individuals and society over the life course. It positions older adults not as passive recipients of age-related change but as active participants who both influence and are influenced by the social systems around them.

Pioneered by Riley et al. [10], the theory rests on five foundational principles. First, individuals move through society in age-based cohorts that experience shared biological, psychological, and social development. Second, each new cohort enters society with a distinct historical and cultural context, shaping its experiences and expectations of ageing. Third, society is organized and stratified by age, with roles and responsibilities often assigned according to age-related norms. Fourth, both individuals and societal roles evolve over time, necessitating ongoing adaptation. Fifth, the relationship between ageing individuals and societal structures is dynamic, characterized by continuous mutual influence.

This framework highlights how generational shifts (such as those resulting from changes in education, technology, or economic conditions) can produce markedly different experiences of ageing among successive cohorts. It also underscores the role of societal institutions in shaping the opportunities and constraints that individuals face as they age.

Subsequent research has reinforced and expanded upon the theory. Uhlenberg [12], for instance, utilized age stratification to explore how social policy can be tailored to meet the distinct needs of ageing cohorts. Yin and Lai [13] examined how variations in cohort histories affect the social statuses and roles of older adults. The theory has also informed studies on residential arrangements, illustrating how generational composition within living environments can impact social interaction, health outcomes, and quality of life [14, 15].

In sum, the Age Stratification Theory offers a nuanced lens through which to examine ageing as a socially constructed process shaped by historical context, cohort dynamics, and institutional frameworks. It reinforces the idea that ageing is not only a biological phenomenon but also a deeply social one, requiring responsive policies that reflect generational diversity and structural change.

### Person-environment fit theory of ageing

The Person–Environment Fit Theory, originally articulated by Lawton [11], offers a foundational framework for understanding the dynamic interaction between an individual’s capabilities and the environmental context in which they function. Central to this theory is the proposition that successful ageing depends on the alignment or “fit” between a person’s competencies and the demands or supports present in their physical and social environment.

According to Lawton, individuals possess a range of personal competencies, including cognitive functioning, sensory and motor skills, psychological resilience, and biological health. These competencies influence how effectively individuals can interact with and adapt to their surroundings. As people age, however, these capacities often decline, rendering them more sensitive to environmental challenges. In such cases, environments that were once manageable may become sources of stress, leading to diminished autonomy, decreased quality of life, or social withdrawal.

The theory posits that when the environment places excessive demands on an older adult whose competencies have declined, a misfit occurs, which may result in feelings of helplessness, frustration, or disengagement. Conversely, environments that are supportive and accommodating (through accessible infrastructure, adaptive technologies, or social support) can buffer against these losses and promote continued engagement and well-being. Therefore, creating environments that adapt to the changing functional abilities of older adults is essential for enhancing their independence and quality of life.

Empirical studies have reinforced the utility of the Person–Environment Fit Theory. For instance, Wahl [16] developed conceptual models to explore how environmental modifications can mitigate age-related challenges, while O’Connor and Vallerand [17] investigated how motivational styles interact with environmental conditions to influence the well-being of older adults in long-term care facilities. These studies underscore the importance of tailoring environments (both physical and psychosocial) to meet the evolving needs of ageing populations.

In summary, the Person–Environment Fit Theory emphasizes that ageing outcomes are not solely determined by intrinsic biological decline but are also profoundly shaped by the compatibility between individuals and their environments. This perspective holds significant implications for designing age-friendly communities, care facilities, and public policies that enable older adults to age with dignity, autonomy, and enhanced well-being.

### Conceptual framework

**Fig. 1:**
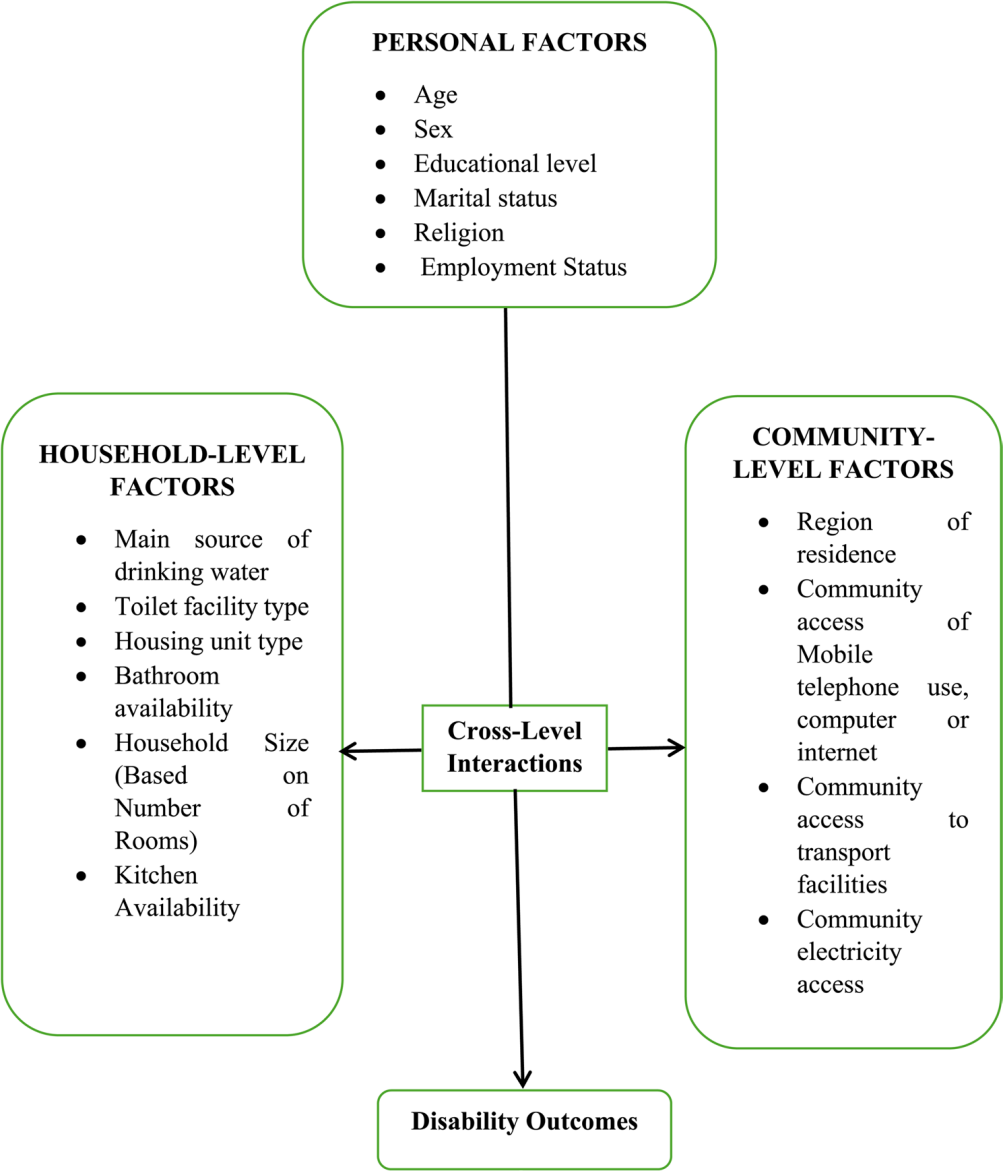
Conceptual framework for understanding disability outcomes among older adults

Drawing from these theoretical perspectives, the framework **(**Fig. 1**)** hypothesizes that disability among older adults in Botswana is shaped by an interplay of personal, household, and community-level factors, each operating independently and interactively. In the diagram, Cross-level interactions refer to situations in which variables from different levels of analysis combine or interact to influence an outcome in ways that are more complex than when each variable acts independently.

At the personal level, several characteristics have been identified as influential. Age is a critical factor, as older individuals, particularly those aged 75 and above, are more likely to experience physical and cognitive decline leading to disability [18]. Gender also matters: women tend to live longer than men but face a higher prevalence of disability, partly due to cumulative socioeconomic disadvantages and caregiving burdens [19]. Educational attainment influences health literacy and the capacity to manage chronic conditions effectively [20], while marital status has both protective and adverse implications. Although being married may provide emotional and social support, potentially reducing disability risk [21], marriage may also serve as a source of stress or abuse in older age, especially for women, thereby contributing to physical or psychological impairments. Religion, as a source of identity and community integration, may contribute to psychological resilience and improved coping, while employment status influences both economic security and social engagement, two key defenses against functional decline.

At the household level, the living environment plays a vital role in determining exposure to health risks and access to supportive infrastructure. Access to safe drinking water is essential for preventing hygiene-related diseases [22], while adequate sanitation (e.g., flush toilets versus pit latrines) is especially crucial for individuals with mobility limitations [23]. The type and quality of housing—such as traditional versus modern structures—can either support or hinder physical independence [24]. Bathroom availability within the household improves personal hygiene, especially for individuals with functional impairments, while overcrowded conditions, reflected by small household size or limited rooms, can elevate stress levels, increase fall risk, and compromise privacy. Likewise, kitchen access contributes to nutritional adequacy, which is vital for sustaining physical strength in old age.

Community-level factors encompass broader infrastructural and geographic contexts that shape access to resources and health-promoting environments. Region of residence—urban, urban village, or rural—plays a significant role in determining access to healthcare, social services, and assistive infrastructure [25]. Urban areas generally provide better access to these resources, whereas rural settings may expose older adults to structural barriers such as transportation difficulties and limited medical services. Digital inclusion (measured by access to mobile phones, computers, and the internet) is increasingly important for older adults to remain informed, socially connected, and able to access telehealth services [26]. Transportation availability, whether through private vehicles, public buses, or non-motorized means, facilitates independence and health service utilization. Electricity access, although often overlooked, supports safety (e.g., lighting for mobility), enables use of medical equipment, and contributes to better housing conditions overall.

Importantly, this framework also emphasizes cross-level interactions. For example, personal factors like low education may amplify the effects of poor community infrastructure, as individuals with limited literacy may be less able to navigate complex healthcare systems or digital platforms. Similarly, the combination of household disadvantage (e.g., lack of indoor plumbing) and community deficits (e.g., absence of reliable transport) can compound barriers to healthcare access and self-care. Conversely, community-level strengths (such as digital access or urban residency) may safeguard personal vulnerabilities. For instance, an older adult with mobility limitations may remain functionally independent if they reside in a digitally connected community with home-based care services.

In sum, the conceptual framework is grounded in the hypothesis that a comprehensive understanding of disability among older adults requires the integration of personal, household, and community-level determinants. It proposes that these domains interact in complex ways, often reinforcing or mitigating each other’s effects, and that disability emerges not solely from biological decline but from the mismatch between an individual’s capacities and their surrounding environment.

### Literature review

Disability in later life represents a growing public health concern, particularly in low- and middle-income countries (LMICs) experiencing demographic transitions. Globally, studies have demonstrated that the prevalence and severity of disability among older adults are influenced by individual, household, and community-level factors. While most research has been conducted in high-income countries, recent studies from LMICs have begun to shed light on context-specific determinants and the structural inequities that shape disability trajectories in ageing populations.

A longitudinal study in India by Paul et al. [27] demonstrated that the incidence and recovery from disability among older adults are strongly influenced by socioeconomic status, education, chronic health conditions, and household living conditions. Notably, individuals with lower education and poor self-rated health were significantly more likely to acquire disability and less likely to recover, suggesting that disability transitions are socially patterned and context-sensitive. Similarly, Ahmad et al. [28], using data from Malaysia’s National Health and Morbidity Survey, identified age, lower education, rural residence, and the presence of chronic diseases as primary predictors of disability in adults, reinforcing the salience of both personal and geographic determinants.

In a broader systematic review, Banks et al. [29] found that poverty both contributes to and is exacerbated by disability across LMICs. Their review confirmed that individuals with disabilities often experience entrenched economic disadvantage, limited access to services, and social exclusion, resulting in a cycle of vulnerability and marginalization. This bidirectional link between poverty and disability underscores the importance of household-level indicators such as income, housing quality, and access to sanitation in understanding the full scope of functional limitations in later life.

Beyond personal and household domains, other studies have emphasized the role of community-level factors. Sousa et al. [30] highlights that chronic conditions such as dementia, stroke, and depression significantly contribute to functional decline in older adults in resource-constrained settings, where community healthcare infrastructure is often inadequate. Additionally, socioeconomic status and neighbourhood disadvantage have been shown to impact both the onset and progression of disability [31].

These studies support the need for integrative, multilevel analyses that consider not only biological ageing but also the structural, social, and environmental conditions that contribute to disability. However, most existing literature either focuses on isolated determinants or single-level analyses. This creates a gap in understanding how these levels interact, particularly in under-researched sub-Saharan African contexts like Botswana.

The present study addresses this gap by employing a multilevel conceptual framework and generalized linear modelling to examine how personal, household, and community factors independently and interactively predict disability severity among older adults. By using a nationally representative dataset and focusing on interaction effects, this study advances the literature by offering a holistic and contextually grounded understanding of disability among older persons in Botswana. The findings contribute to a growing body of work that advocates for policy responses that are both inclusive and multisectoral, targeting not only individual risk but also household and infrastructural inequities that shape the lived experience of disability.

## Methods

### Data, study design, and sample

The analysis in this paper was based on data from the 2022 Botswana Population and Housing Census (PHC). The 2022 PHC utilized the Washington Group Short Set (WGSS) of questions to measure disability. The WGSS, developed by the United Nations City Group, is designed to identify individuals who are at risk of participation restrictions due to health conditions or impairments. The tool focuses on six fundamental functional domains: seeing, hearing, walking, cognition, communication, and self-care. The data relevant to this study was drawn from individuals aged 65 years and older. The sample of the study was 95 890 older adults extracted from the 2022 Population and Housing Census. This data source provided a national comprehensive basis for investigating the influence of individual, household, and community-level factors on the prevalence of disabilities among older adults in Botswana.

### Measurement of outcome variable: old-age disability count

To construct a parsimonious and analytically coherent measure, the study operationalizes the outcome variable (old-age disability count) based on three core domains derived from the 2022 Botswana Population and Housing Census:

- Mobility: Difficulty walking or climbing steps.
- Functional Self-Care: Difficulty performing activities of daily living, such as bathing, dressing, or feeding.
- Cognitive: A composite domain that captures difficulties in remembering or concentrating and/or difficulty in communicating clearly.

The decision to merge “remembering/concentrating” and “communicating” into a unified cognitive domain is theoretically grounded in geriatric and neurological literature, where these dimensions often co-occur and are mutually reinforcing. In conditions such as dementia or mild cognitive impairment, deficits in memory are frequently accompanied by expressive or receptive communication challenges [32]. Thus, the combined indicator captures a more holistic measure of cognitive functioning and reflects established practice in similar empirical analyses, particularly when working within census-based data constraints.

Each domain is dichotomized using a threshold-based coding scheme: respondents who reported “some difficulty,” “a lot of difficulty,” or “cannot do at all” in each domain were assigned a score of 1, while those reporting “no difficulty” received a score of 0. The sum of scores across the three domains yields an individual-level disability count ranging from 0 to 3, where higher scores reflect greater levels of functional impairment.

Although the original Washington Group Short Set (WG-SS) includes up to six distinct functional domains, the decision to focus on these three was informed by the conceptual coherence of the chosen domains, their empirical reliability in older populations, and the structure of the available census data. This tailored approach maintains methodological transparency, aligns with internationally recognized disability assessment frameworks, and supports rigorous inferential analysis.

While the theoretical range of the disability count variable is capped at three, preliminary analyses were conducted to examine the empirical distribution of scores and assess whether the selected domains capture meaningful distinctions in functional status. Descriptive statistics, including the observed range, mean, and variance of the disability count, were presented in the results section. The old-age disability count, as constructed, offers a succinct yet robust proxy for functional health and dependency among individuals aged 65 and older, supporting subsequent analysis of its demographic and socioeconomic determinants.

### Model selection strategy

This study aimed to investigate the determinants of disability among older adults in Botswana by examining the complex interplay between individual, household, and community-level factors. Given this multi-layered objective, the adoption of a full model incorporating all relevant covariates across levels was critical.

To determine the most appropriate count regression model for the outcome variable (old-age disability count), two candidate models were considered: the Poisson regression model and the Negative Binomial (NB) regression model. The Poisson model is traditionally used for count data under the assumption of equilibrium dispersion, where the mean and variance of the dependent variable are approximately equal. However, this assumption often does not hold in real-world data, especially in large, heterogeneous populations where overdispersion (variance exceeding the mean) is common. Overdispersion can lead to underestimation of standard errors and inflated Type I error rates in Poisson models, thus compromising the reliability of results [33].

The selection of the best-fitting model was guided by several statistical diagnostics. First, deviance and Pearson goodness-of-fit statistics were examined. Deviance values close to 1 suggest that the Poisson model is suitable; however, values exceeding 1.2 may indicate overdispersion, prompting consideration of the NB model as a more appropriate alternative [34]. Second, model performance was compared using Akaike’s Information Criterion (AIC) and the Bayesian Information Criterion (BIC). Both metrics balance model fit and complexity, with lower values indicating a better-fitting and more parsimonious model [35, 36]. BIC applies a stricter penalty for additional parameters, offering a conservative benchmark for model selection. Third, a Likelihood Ratio Chi-Square Test was used to assess whether the added dispersion parameter in the NB model provides a statistically significant improvement over the Poisson model. A p-value below 0.05 indicated that the more flexible model better captures the data structure.

By applying the deviance, AIC, BIC, and the Likelihood Ratio Test, the most appropriate model was selected for the final analysis. This data-driven approach ensures the chosen model is not only statistically sound but also well-suited to capturing the complexity of disability among older adults in Botswana, particularly considering the study’s goal to incorporate individual, household, and community-level determinants.

### Statistical analysis

To assess the severity of disabilities among older adults in Botswana, descriptive statistics were employed, providing an initial overview of the distribution and extent of old-age disabilities. At the multivariate level, generalized linear models (GLMs) were utilized to examine the personal, household, and community-level determinants of disabilities among older adults. By analysing determinants across multiple levels, this study offers a comprehensive understanding of the complex factors influencing disability prevalence. The results are presented as incidence rate ratios (IRRs), coefficients and standard errors. A 5% significance level (*p* < 0.05) was used to identify statistically significant predictors.

All statistical analyses were conducted using IBM SPSS Statistics (Version 29.0). While R and Stata are commonly used in population health research, SPSS was selected due to its reliable capacity for handling large-scale census microdata and for its comprehensive implementation of GLM procedures. Nonetheless, future extensions of this work may consider alternative statistical packages to enhance computational flexibility and reproducibility.

## Results

### Individual-level characteristics of the study population

Table 1 below shows that the study population consists of 95,890 older adults, with a relatively balanced distribution across different age groups. The highest proportion of participants were aged 65–69, comprising 35.5% of the population, followed by the 80 + age group, which makes up 24.4%. The smallest proportion of participants were individuals aged 75–79, accounting for 16.0%, while those aged 70–74 represent 24.2% of the total population. In terms of gender, the population was slightly skewed towards females, who represent 55.6% of the group, while males account for 44.4%. Regarding education levels, a significant proportion of the older adults, 34.8%, had primary-level education or less. Only 13.4% had completed secondary education, and 11.7% have attained tertiary or higher education. With respect to marital status, 34.8% of the older adults were currently married, and a similar proportion, 34.5%, were never married. A smaller segment, 3.7%, were those living together without formal marriage, while 27.0% had previously been married (divorced, widowed, or separated). The religious affiliation of the population was predominantly Christianity, with 85.3% identifying as Christians. 6.5% adhere to African Traditional Religion, while 7.0% reported no religious affiliation, and 1.1% follow other religions. The employment status revealed that majority of older adults, 77.7%, were not employed, indicating a large retired or economically inactive population. Only 22.3% were employed, reflecting the limited workforce participation among this age group, which is expected given the typical retirement age.

**Table 1:** Individual-level characteristics of the study population

Characteristics	Frequency	Percentage
	*N* = 95,890	100%
Age-group		
65–69	34,006	35.4
70–74	23,173	24.2
75–79	15,344	16.0
80+	23,367	24.4
Sex		
Male	42,616	44.4
Female	53,274	55.6
Education		
Primary/less	39,245	74.9
Secondary	7022	13.4
Tertiary/higher	6128	11.7
Marital status		
Married	33,292	34.8
Never married	33,010	34.5
Living together	3531	3.7
Previously married	25,786	27.0
Religion		
Christianity	81,827	85.3
African Tradition Religion	6260	6.5
No religion	6706	7.0
Others	1097	1.1
Employment status		
Employed	21,298	22.3
Not employed	74,319	77.7

### Household-level characteristics of the study population

The household-level characteristics of the study population provide valuable insights into living conditions and infrastructure access among the 95,890 older adults in the study population. In terms of drinking water sources, 28.0% of households had access to piped water indoors, while the largest proportion, 45.0% relied on piped water outdoors. Additionally, 27.0% of households used other sources of drinking water, which include wells, boreholes, or rivers. Regarding sanitation, the most common type of toilet facility is the pit latrine, used by 53.7% of households. 31.6% of households had a flush toilet, while 1.6% used dry compost toilets. Notably, 13.1% of households did not have any toilet facility. With respect to bathroom availability, 41.7% of households had a bathroom within their housing units. However, the majority, 53.9%, lack a bathroom altogether. A small proportion, 4.4%, had a bathroom located outside the housing unit. The household size based on the number of rooms showed that 43.5% of households were medium-sized, with 3–4 rooms, while 38.0% were small-sized households with only 1–2 rooms. 18.5% of households were classified as large, having 5 or more rooms. Regarding kitchen availability, just over half of the households, 51.4%, had an indoor kitchen. 16.3% of households had an indoor cooking space that is not a designated kitchen, and 17.1% rely on outdoor cooking spaces. Notably, 15.2% of households lack any designated cooking space. The results are presented in Table 2.

**Table 2:** Household-level characteristics of the study population

Characteristics	Frequency	Percentage
	*N* = 95,890	100%
Main source of drinking water		
Piped indoors	26,861	28.0
Piped outdoors	43,112	45.0
Other sources	25,906	27.0
Toilet facility type		
Flush toilet	30,274	31.6
Pit-latrine	51,491	53.7
Dry compost toilet	1546	1.6
No toilet facility	12,567	13.1
Housing unit type		
Traditional housing	8426	8.8
Mixed housing	29,350	30.6
Modern housing	55,411	57.8
Other housing types	2697	2.8
Bathroom availability		
Available within housing unit	39,991	41.7
Available outside housing unit	4198	4.4
No bathroom available	51,689	53.9
Number of rooms in a Household		
Small-sized Household (1–2 rooms)	36,283	38.0
Medium-sized household (3–4 rooms)	41,512	43.5
Large-sized household (5 + rooms)	17,659	18.5
Kitchen availability		
Indoor kitchen	49,302	51.4
Indoor cooking space (non-kitchen)	15,602	16.3
Outdoor cooking space	16,360	17.1
No designated cooking space	14,614	15.2

### Community-level characteristics of the study population

The community-level characteristics of the study population, comprising 95,890 older adults, highlight significant differences in infrastructure and access to essential services across various regions. Regarding the region of residence, most of the population, 51.0%, lived in rural areas, while 41.3% lived in urban villages. Only 7.7% of the study population lives in cities and towns. In terms of mobile phone access, 81.8% of the population had access to mobile phones. However, 18.2% did not have access to mobile phones. With respect to computer use, 6.3% of the population had access to computers, while a 93.7% did not. Regarding internet accessibility, 10.7% of the population had access to the internet. Majority, 89.3%, did not have internet access. The transportation infrastructure in the community showed that 61.0% of the population relied on public transportation as their main mode of transport. 22.3% had access to private vehicles, while 9.0% relied on non-motorized transport such as walking or bicycles. Notably, 7.8% of the population had no access to transportation at all. In terms of electricity access, 65.1% of the population were connected to the national electrical grid, while 34.9% relied on off-grid sources such as solar panels or generators. The results are presented in Table 3.

**Table 3:** Community-level characteristics of the study population

Characteristics	Frequency	Percentage
	*N* = 95,890	100%
Region of residence		
Cities and towns	7425	7.7
Urban villages	39,580	41.3
Rural areas	48,885	51.0
Community access to mobile-telephone use		
Have access to mobile telephone	78,161	81.8
Does not have access to mobile telephone	17,443	18.2
Community access to computer use		
Have access to computer use	5996	6.3
Does not have access to computer use	89,608	93.7
Community access to internet		
Have access to internet	8375	10.7
Does not have access to internet	69,931	89.3
Community access to transport facilities		
Private vehicle	21,297	22.3
Public transport	58,285	61.0
Non-motorized transport	8584	9.0
No transport	7438	7.7
Community electricity access		
National electrical grid	62,440	65.1
Off-grid sources	33,438	34.9

### Distribution and severity of disabilities among older adults

**Fig. 2:**
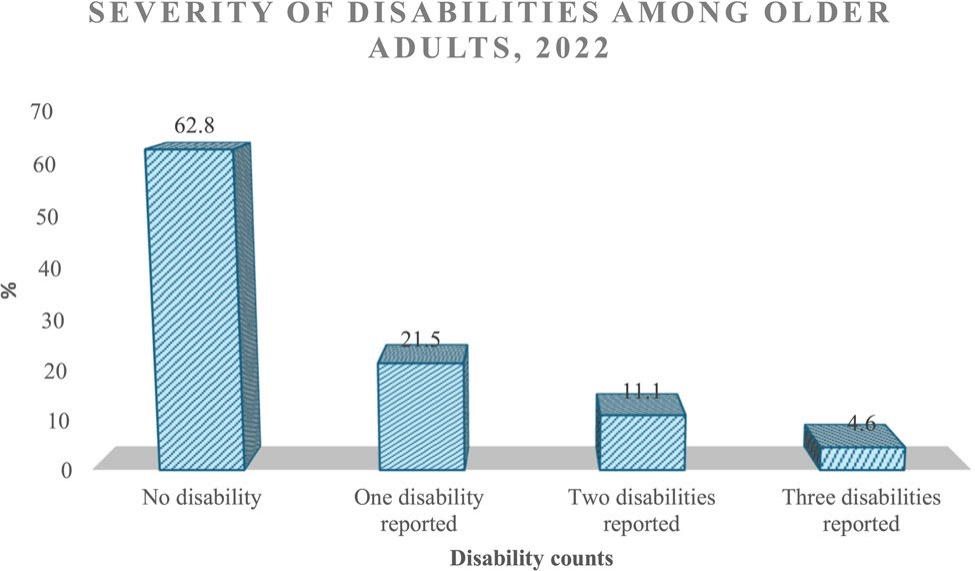
Severity of disabilities among older adults

The chart in Fig. 2 presents the distribution of disability counts among older adults, categorized by the number of disabilities reported. The data is displayed as percentages, offering insight into the prevalence and severity of disabilities within this population.

Most older adults, 62.8%, reported no disabilities. This suggests that many older adults maintain a relatively high level of physical and cognitive functioning. However, a notable portion, 21.5%, reported experiencing one disability, representing a shift toward some level of functional limitation among a substantial segment of the population. This single disability may range from mobility impairments to functional or cognitive challenges.

The percentage of older adults who reported two disabilities stood at 11.1%, reflecting the cumulative burden of multiple impairments on daily life. The decline in this figure compared to the previous category suggests that while some individuals experience multiple disabilities, the prevalence of such cases is lower. Finally, the percentage of individuals reporting three disabilities stood at 4.6%, representing the most severe end of the disability spectrum among older adults for this study. This small yet significant group likely faces more complex challenges in health management and daily functioning.

While only 4.6% of older adults in the sample reported having three disabilities, this small proportion does not pose a methodological concern because the outcome variable is modelled as a count rather than a set of predefined categories. The key point is that the count models estimate a relationship between covariates and the rate or expected number of occurrences of the outcome (i.e., number of disabilities), rather than estimating parameters for each specific count level. Thus, even relatively rare count values, such as 3 disabilities, are retained as part of the full count spectrum and contribute to the estimation process. Provided that these higher counts are not extreme outliers and occur with reasonable consistency, they do not threaten model stability.

Furthermore, treating the variable as a continuous count rather than collapsing it into broad categories preserves the granularity and richness of the data, avoids arbitrary thresholds, and improves statistical efficiency. For instance, knowing that someone has 3 disabilities provides more detail than simply knowing they “have a disability”.

### Descriptive statistics of the outcome variable

The descriptive statistics of the old-age disability count variable, derived from a large sample of 95,890 individuals, reveal important insights into the functional health profile of older adults in Botswana. The observed range of disability counts spans from 0 to 3, aligning precisely with the study’s operationalization of disability across three core functional domains: mobility, functional self-care, and cognitive disability. A minimum score of 0 reflects individuals who reported no functional limitations in any domain, while the maximum score of 3 indicates the presence of some level of difficulty across all three domains. The results are presented in Table 3.

The mean disability score is 0.574, indicates that, on average, older adults in Botswana report slightly more than half of one functional difficulty, with a substantial proportion likely experiencing no disability or only one form of limitation. The relatively high standard deviation of 0.86105, in relation to the mean, further indicates considerable variability in disability experiences among this population group. This dispersion suggests that while many older adults remain functionally independent, others contend with more pronounced functional challenges.

The variance of 0.741 reinforces this observation of heterogeneity, highlighting the uneven burden of disability within the ageing population. Taken together, these findings validate the study’s use of three functional domains, which not only reflect conceptually significant aspects of disability in later life but also yield sufficient variability for robust statistical analysis. The distribution of scores confirms that the selected domains capture meaningful distinctions in functional status among older adults in Botswana, thus providing a solid foundation for examining the determinants and implications of disability in this demographic group Table 4.

**Table 4:** Descriptive statistics of old age disability count variable

Variable	Range Statistic	Minimum Statistics	Maximum Statistics	Mean Statistic	Mean Std. Error	Standard Deviation	Variance Statistic
Old Age Disability Count	3.00	0.00	3.00	0.5744	0.00278	0.86105	0.741

### Model selection and justification

Table 5 below illustrates the evaluation of multiple goodness-of-fit statistics, including deviance, Akaike’s Information Criterion (AIC), Bayesian Information Criterion (BIC), and likelihood-based significance tests. The aim was to determine the most appropriate model to analyse the relationship between individual, household, and community-level determinants of disability among older adults in Botswana.

**Table 5:** Model fit statistics for Poisson vs. Negative binomial regression models (Models 0–4)

Poisson Regression Model					
**Goodness of Fit Statistics**	**Model 0** (Empty Model)	**Model 1** (Individual level characteristics)	**Model 2** (Household level Characteristics**)**	**Model 3** (Community level characteristics)	**Model 4** (Full Model)
Deviance (value/df)	1.247	1.043	1.236	1.135	1.002
Akaike’s Information Criterion (AIC)	201,134	95,039	199,119	149,170	81,568
Finite Sample Corrected AIC (AICC)	201,134	95,039	199,119	149,170	81,568
Bayesian Information Criterion (BIC)	201,144	95,163	199,271	149,263	81,901
Consistent AIC (CAIC)	201,145	95,177	199,271	149,273	81,939
Likelihood Ratio Chi-Square (sig.)	-	< 0.001	< 0.001	< 0.001	< 0.001
Wald Chi-Square (sig)	< 0.001	< 0.001	< 0.001	< 0.001	< 0.001

Negative Binomial Regression Model					
Goodness of Fit Statistics	Model 0	Model 1	Model 2	Model 3	Model 4
Deviance (value/df)	0.840	0.726	1.236	0.788	0.708
Akaike’s Information Criterion (AIC)	197,524	95,631	199,119	147,625	82,075
Finite Sample Corrected AIC (AICC)	197,524	95,631	199,119	147,625	82,075
Bayesian Information Criterion (BIC)	197,533	95,755	199,271	147,718	82,408
Consistent AIC (CAIC)	197,534	95,769	199,271	147,728	82,446
Likelihood Ratio Chi-Square (sig.)	-	< 0.001	< 0.001	< 0.001	< 0.001
Wald Chi-Square (sig)	< 0.001	< 0.001	< 0.001	< 0.001	< 0.001

Among the five sequential models tested under both frameworks (Models 0 to 4), Model 4 was the most comprehensive, incorporating individual, household, and community-level covariates. This specification aligns with the study’s overarching goal of adopting a multilevel and holistic approach to understanding disability in later life. In the Poisson regression framework, Model 4 yielded a deviance-to-degrees-of-freedom ratio of 1.002, which is closest to the ideal value of 1, indicating an excellent model fit and minimal overdispersion. Furthermore, Model 4 recorded the lowest values across all information criteria (AIC (81,568), AICC (81,568), BIC (81,901), and CAIC (81,939)) compared to other Poisson and Negative Binomial specifications. These results suggest that, when all relevant predictors are included, the Poisson model adequately captures the variation in the data without the need for an additional dispersion parameter.

Although the Negative Binomial models also demonstrated good fit, especially in earlier stages where overdispersion was more apparent, the superior performance of Poisson Model 4 justifies its selection for the final analysis. The model not only meets statistical assumptions but also offers clearer interpretability and parsimony, which are valuable for informing policy recommendations. Importantly, by incorporating variables from all three levels (individual, household, and community) Poisson Model 4 enables a nuanced understanding of the multifaceted nature of disability. This multilevel integration is essential for crafting inclusive and targeted interventions that address the structural, familial, and personal dimensions of ageing and disability in Botswana.

Therefore, based on statistical rigor, theoretical coherence, and alignment with the study’s policy-oriented objectives, the Poisson regression Model 4 is identified as the best-fitting model and is adopted for the main analysis.

### Poisson regression analysis of determinants of disability prevalence among older adults in Botswana

Table 6 presents the results of a Poisson regression model examining the individual, household, and community-level determinants associated with the severity of disability among older adults in Botswana. The dependent variable is the count of functional disabilities, and the model controls for a wide range of sociodemographic and environmental factors. The model is statistically significant overall (LR χ² = 5877.43, *p* < 0.05), with a large sample size (*N* = 47,309), indicating robust analytical power. All coefficients are presented alongside standard errors and incidence rate ratios (IRRs), with 95% confidence intervals provided to enhance interpretability.

**Table 6:** Poisson regression of factors influencing the severity of old age disability in Botswana

Characteristics	Coefficients	Standard Error	IRR
**Interaction effects**	Full model (95% CI)	Full model	Full model (95% CI)
Age-group			
65–69	−0.835 (−872; −0.798)	0.0188	0.434* (0.418; 0.450)
70–74	−0.0551(−0.588; −0.513	0.0191	0.576* (0.555; 0.598)
75–79	−0.317 (−0.356; −0.277)	0.0203	0.729* (0.700; 0.758)
80+	0		1
Sex			
Male	−0.433 (−0.470; −0.397	0.0186	0.648* (0.625; 0.672)
Female			1
Education			
Primary/less	0.188 (0.128; 0.248)	0.0307	1.207* (1.136; 1.282)
Secondary	0.063 (−0.005; 0.130)	0.0344	1.065 (0.995; 1.139)
Tertiary/higher	0		1
Marital status			
Married	−0.050 (−0.087; −0.013)	0.0189	0.951* (0.917; 0.987)
Never married	−0.088 (−0.121; −0.055)	0.0169	0.916* (0.886; 0.947)
Living together	0.032 (−0.064; 0.128)	0.0488	1.032 (0.938; 1.136)
Previously married	0		1
Religion			
Christianity	−0.079 (−0.219; 0.062)	0.0718	0.924 (0.803; 1.064)
African Tradition Religion	0.016 (−0.141; 0.172)	0.0798	1.016 (0.869; 1.188)
No religion	−0.071 (−0.228; 0.087)	0.0804	0.932 (0.796; 1.091)
Others	0		1
Employment status			
Employed	−0.354 (−0.391;−0.316)	0.0191	0.702* (0.676; 0.729)
Not employed	0		1
Main source of drinking water			
Piped indoors	−0.031 (−0.090; 0.027)	0.0298	0.969 (0.914; 1.027)
Piped outdoors	−0.035 (−0.081; 0.011)	0.0234	0.965 (0.922; 1.011)
Other sources	0		1
Toilet facility type			
Flush toilet	0.051 (−0.033; 0.134)	0.0427	1.052 (0.968; 1.144)
Pit latrine	0.015 (−0.056; 0.085)	0.0359	1.015 (0.946; 1.089)
Dry compost toilet	−0.093 (−0.260; 0.073)	0.848	0.911 (0.771; 1.076)
No toilet facility	0		1
Housing type unit			
Traditional housing	0.026 (−0.112; 0.164)	0.0703	1.026 (0.894; 1.178)
Mixed housing	0.013 (−0.120; 0.147)	0.0682	1.013 (0.887; 1.158)
Modern housing	−0.016 (−0.150; 0.119)	0.0686	0.985 (0.861; 1.126)
Other housing types	0		1
Bathroom availability			
Available within housing unit	−0.009 (−0.051; 0.034)	0.0216	0.991 (0.950; 1.034)
Available outside housing unit	0.016 (−0.054; 0.086)	0.0358	1.016 (0.947; 1.090)
No bathroom available	0		1
Number of rooms in a Household			
Small-sized household (1–2 rooms)	0.089 (0.048; 0.130)	0.0210	1.093* (1.049; 1.139)
Medium-sized household (3–4 rooms)	0.046 (0.011; 0.081)	0.0179	1.047* (1.011; 1.084)
Large-sized household (5 + rooms)	0		1
Kitchen availability			
Indoor kitchen	0.017 (−0.037; 0.072)	0.0277	1.018 (0.964; 1.074)
Indoor cooking space (non-kitchen)	−0.038 (−0.094; 0.018)	0.0287	0.963 (0.910; 1.018)
Outdoor cooking space	−0.030 (−0.088; 0.027)	0.0294	0.970 (0.916; 1.028)
No designated cooking space	0		1
Region of residence			
Cities and towns	−0.137 (−0.193; 0.081)	0.0285	0.872* (0.825; 0.922)
Urban villages	−0.091 (−0.122; 0.059)	0.0162	0.913* (0.885; 0.943)
Rural areas	0		1
Community access to mobile phones			
Have access to mobile phones	−0.254 (−0.638; 0.129)	0.1958	0.775 (0.528; 1.138)
Does not have access to mobile phone	0		1
Community access to computer use			
Have access to computer use	−0.369 (−0.447; −0.291)	0.0398	0.692* (0.640; 0.748)
Does not have access to computer use	0		1
Community access to internet			
Have access to internet	−0.259 (−0.323; −0.195)	0.0326	0.772* (0.724; 0.823)
Does not have access to internet	0		1
Community access to transport facilities			
Private vehicle	−0.114−0.178; −0.050)	0.0327	0.892* (0.837; 0.951)
Public transport	−0.117 (−0.175; −0.060)	0.0294	0.889* (0.840; 0.942)
Non-motorized transport	−0.103 (−0.183; −0.023)	0.0406	0.902* (0.833; 0.977)
No transport	0		1
Community access to electricity			
National electrical grid	−0.103 (−0.149; −0.057)	0.0235	0.902* (0.862; 0.945)
Off-grid sources	0		1
N	47,309		
LR Chi2	5877.43*		
Log Likelihood	−40746.42		

Source: Authors’ estimates based on the 2022 PHC data

Significance levels: ^*^p <0.05

Age group emerged as a significant predictor, showing a clear inverse gradient: individuals aged 65–69 and 70–74 had significantly lower expected disability counts compared to those aged 80 and above (reference group), with IRRs of 0.43 and 0.58, respectively. Interestingly, individuals aged 75–79 also showed a reduced, albeit less pronounced, risk (IRR = 0.73), suggesting that functional impairments increase more sharply beyond age 80. Males were significantly less likely than females to report higher disability counts (IRR = 0.65, *p* < 0.05), a finding that aligns with prior literature on gendered health disparities in older populations.

Educational attainment was strongly associated with reduced disability. Older adults with tertiary or higher education served as the reference group, and those with primary education or less exhibited a 20.7% higher incidence of disability (IRR = 1.207, *p* < 0.05). While secondary education showed a positive coefficient, it was not statistically significant. These findings suggest that lower education levels may be linked to diminished health literacy, lifelong occupational exposures, or reduced access to healthcare, all of which influence disability risk in later life.

Marital status was also significant, with currently married individuals and those who had never married exhibiting lower disability counts compared to those previously married (widowed, divorced, or separated). For instance, never-married individuals showed an IRR of 0.916 (*p* < 0.05), indicating a potential protective effect associated with marital status, possibly mediated by social support networks.

Employment status was another strong predictor: employed older adults had 30% lower expected disability counts than their unemployed counterparts (IRR = 0.702, *p* < 0.05), highlighting the health benefits of continued economic engagement or selection effects whereby healthier individuals remain employed longer.

At the household level, small and medium-sized households (based on number of rooms) were significantly associated with increased disability risk, possibly reflecting overcrowding or resource constraints. By contrast, variables such as access to piped water, toilet type, bathroom availability, and kitchen location did not show statistically significant associations, indicating that these basic amenities, while important, may not independently influence disability counts once other factors are controlled for.

The region of residence was a significant predictor: older adults living in cities and towns (IRR = 0.872, *p* < 0.05) and urban villages (IRR = 0.913, *p* < 0.05) had significantly lower disability counts compared to their rural counterparts, underscoring persistent geographic inequities in access to health services and supportive environments.

Community-level indicators further reinforced the role of structural determinants. Access to computers (IRR = 0.692, *p* < 0.05), internet (IRR = 0.772, *p* < 0.05), and transportation (whether private, public, or non-motorized) were all significantly associated with lower disability counts. Notably, older adults in communities with national grid electricity access had a 10% reduction in disability (IRR = 0.902, *p* < 0.05), pointing to the broader role of infrastructure and technological access in promoting healthy ageing.

Although not explicitly labelled, the model appears to account for interaction effects by examining how multiple variables across levels (individual, household, and community) influence disability severity in a nested manner. For instance, the combined effects of region and infrastructure (e.g., electricity, internet) may reflect broader environmental moderators of health outcomes among older adults.

Overall, the model affirms that the severity of old-age disability in Botswana is shaped by a combination of demographic, socioeconomic, and environmental factors. The evidence underscores the need for multi-sectoral policy responses that integrate health, education, infrastructure, and social protection to support healthy ageing across diverse contexts.

## Discussion

The Poisson regression full model, which integrates individual, household, and community-level determinants, provided the best statistical fit and most comprehensive insight into the severity of disability among older adults in Botswana. This multilevel specification aligns with both the theoretical underpinnings of the study (including the ICF, Age Stratification, and Person–Environment Fit theories) and its practical aim of informing policy through a contextualized understanding of disability. The results confirm that functional impairment in later life is shaped not only by individual attributes such as age, sex, and education, but also significantly influenced by household living conditions (e.g., housing size, sanitation access) and community infrastructure (e.g., electricity, transport, internet access). The model highlights that reducing disability requires more than medical or behavioural interventions; it demands coordinated, cross-sectoral strategies that improve environmental accessibility, digital inclusion, and rural infrastructure. The findings affirm that ageing with dignity and reduced disability in Botswana depends on policies that address both the social environment and structural conditions that shape health across the life span.

Furthermore, this study reinforces the multidimensional and cumulative nature of disability among older adults in Botswana, where age, gender, education, employment, household context, and community infrastructure converge to shape functional health outcomes. As observed in the Poisson model, age remains a central determinant of disability severity. Individuals aged 65–69 and 70–74 experience significantly lower disability counts compared to those aged 80 and above, suggesting a progressive escalation of functional limitations with advancing age. This aligns with global evidence showing that increasing age is associated with physical decline, frailty, and cognitive deterioration, which together raise the likelihood of disability in late life [37]. The non-linear gradient identified between age sub-groups, with a sharper rise after age 75, underlines the importance of early preventive interventions and age-sensitive health services tailored to the “younger old” and “older old” alike.

Gender disparities in disability are also pronounced, with males exhibiting substantially lower disability counts than females. This result is consistent with existing studies that attribute higher disability prevalence among older women to a combination of biological longevity, lifetime caregiving burdens, lower socio-economic security, and unequal access to healthcare resources [38]. The persistence of these disparities after controlling for socioeconomic and environmental variables points to structural gender inequities that accumulate over the life course. Therefore, gender-responsive health policies (especially those that enhance access to healthcare, social protection, and caregiver support for older women) are essential for reducing the disability burden among this vulnerable group.

Educational attainment emerges as a powerful protective factor. Older adults with primary education or less are significantly more likely to experience disabilities compared to those with tertiary or higher education. This finding is in line with the established social gradient in health, where lower education correlates with poorer health outcomes due to limited health literacy, occupational exposures, and reduced access to information and healthcare services [39, 40]. While secondary education showed a positive coefficient, it was not statistically significant, suggesting that the threshold of benefit may be greatest for those attaining tertiary-level education. These results emphasize the long-term benefits of investing in equitable education systems that foster lifelong health resilience.

Employment status also demonstrates a strong and significant association with disability outcomes. Employed older adults report nearly 30% fewer disabilities than their unemployed counterparts, reaffirming the role of economic engagement in supporting health in later life. Employment not only offers financial stability but also promotes social participation, purpose, and routine, all of which are protective against both physical and mental decline [41]. This underscores the potential of active ageing policies that facilitate flexible, age-friendly employment opportunities and support productive engagement for older persons.

At the household level, individuals living in small (1–2 room) and medium-sized (3–4 room) households experience significantly higher disability counts than those in larger households. This likely reflects the adverse health consequences of overcrowding, such as limited privacy, heightened stress, and constrained mobility within the home environment [42]. While household amenities like toilet type, kitchen availability, and bathroom location did not emerge as significant predictors in the full model, their indirect effects may still be important when considered in interaction with other variables such as housing type, region, and income. For instance, in rural or low-income settings, poor sanitation or lack of indoor facilities could exacerbate mobility limitations or infection risks, particularly for individuals already facing functional impairments.

Geographic disparities also surfaced, with older adults residing in urban villages and cities reporting significantly lower disability counts compared to their rural counterparts. This “urban advantage” likely reflects better access to healthcare facilities, social infrastructure, and supportive services in non-rural settings [43]. However, this urban–rural divide in disability prevalence signals a critical equity issue. Rural areas may suffer from inadequate health infrastructure, transportation barriers, and longer travel distances to health services, all of which restrict the ability of older residents to manage and prevent disability. Targeted resource allocation and decentralized health services are therefore necessary to close these geographic gaps.

Technological infrastructure (particularly access to computers, internet, and electricity) also plays a significant role in reducing disability prevalence. The study finds that older adults living in communities with digital and electrical connectivity experience markedly fewer disabilities. These results support previous findings by the World Bank Group [44], which highlight the transformative potential of information and communication technologies (ICTs) for persons with disabilities across domains such as healthcare, education, and civic engagement. Enhancing digital literacy and infrastructure (especially in rural and underserved communities) can empower older adults to access telehealth services, maintain social networks, and gain health-related information, all of which are essential for maintaining autonomy and functional health.

Transportation access (whether via private vehicles, public transport, or non-motorized means) emerged as a robust predictor of lower disability risk. This finding underscores the vital role of mobility in supporting access to healthcare, community engagement, and independence among older adults [45]. The lack of transport infrastructure can severely limit access to routine care, rehabilitation, and social support networks, thereby exacerbating disability risks. As such, mobility-inclusive urban planning and age-friendly transportation policies should be prioritized, particularly in remote and under-resourced settings.

Finally, although interaction terms were not explicitly modelled, the structure of the multilevel regression and the inclusion of variables across individual, household, and community dimensions allow us to infer several meaningful interactions. For instance, the protective effect of infrastructure (e.g., electricity or internet) may be stronger in rural areas, where baseline access is limited. Likewise, the influence of education or employment may be moderated by gender or marital status, reflecting the layered vulnerabilities and resources that shape ageing experiences. These findings reinforce the relevance of the WHO’s International Classification of Functioning (ICF) model, which conceptualizes disability as a dynamic interaction between health conditions and contextual factors, including personal and environmental influences.

In summary, the determinants of old-age disability in Botswana are multifactorial and intersecting. Addressing them requires integrated strategies that combine healthcare access, educational equity, infrastructural development, gender-sensitive policy design, and community empowerment. Investing in inclusive environments that support active and healthy ageing will be critical to mitigating the burden of disability and promoting wellbeing among Botswana’s growing older population.

## Conclusion

This study provides a comprehensive and empirically grounded understanding of the multifaceted determinants influencing the severity of disability among older adults in Botswana. Drawing on a nationally representative dataset and robust Poisson regression analysis, the findings illuminate how disability in later life is shaped not solely by biological ageing, but by a complex interplay of individual, household, and community-level factors.

Age remains a strong predictor of functional limitation, with disability increasing markedly after age 75, underscoring the progressive nature of health decline in older adulthood. Gender disparities are pronounced, reinforcing the cumulative disadvantage experienced by older women due to intersecting social, economic, and health-related vulnerabilities. Education and employment emerge as powerful protective factors, highlighting the critical importance of life-course investments in human capital to buffer against age-related decline.

Importantly, the study reveals that household living conditions, access to infrastructure, digital connectivity, and mobility resources significantly influence disability outcomes. Older adults residing in better-equipped homes and communities with access to transport, electricity, internet, and computer use experience fewer disabilities, emphasizing the crucial role of the built environment and service availability in shaping ageing trajectories.

The results affirm the relevance of the WHO’s International Classification of Functioning (ICF) framework and are theoretically enriched by the Person–Environment Fit and Age Stratification theories, which together underscore that disability is not only a clinical condition but also a function of how older individuals interact with their environments over time.

Critically, this study calls for an integrated, multisectoral response to ageing and disability—one that combines inclusive health services, gender-sensitive social protection, education, and infrastructure development. Policies should prioritize equity by targeting rural communities, digitally disconnected populations, and those living in substandard housing, ensuring that no older person is left behind.

As Botswana’s older population continues to grow, the imperative is clear: to create inclusive, age-friendly environments that enable functional independence, uphold dignity, and promote healthy, active ageing for all. This requires evidence-informed planning, interministerial collaboration, and sustained investment in both people and the systems that support them. The insights from this study offer a critical roadmap for that transformative agenda.

### Limitations of the chapter

While this study provides valuable insights into the determinants of disability prevalence among older adults in Botswana, several limitations must be acknowledged. First, the use of cross-sectional data from the 2022 Population and Housing Census limits the ability to establish causality. Cross-sectional data limit statements related to temporal causality [46]. The associations identified between the various individual, household, and community-level factors and disability outcomes are correlational, and it is not possible to infer temporal relationships or determine whether certain factors directly cause increased disability. Future research should incorporate longitudinal data to examine how these factors influence disability progression over time.

Second, despite the comprehensive nature of the analysis, the reliance on self-reported measures of disability may introduce reporting bias [47]. Older adults may underreport or overreport their disabilities due to social desirability bias, memory issues, or cultural perceptions of disability. Objective measures of disability, such as medical assessments, could enhance the accuracy of the data and provide a more precise understanding of disability prevalence.

Third, the study’s geographic scope, focusing primarily on urban-rural distinctions, may overlook district heterogeneity within urban and rural areas. Variations in infrastructure, healthcare access, and social support systems across different district within Botswana might result in differential disability experiences, which the study may not fully capture. Future research should explore district disparities in greater detail to identify location-specific interventions that could address unique challenges faced by older adults in different areas.

Moreover, the study’s focus on household and community-level infrastructure, while important, does not fully account for psychological and social factors such as mental health, social isolation, or access to community-based support services, all of which could significantly influence disability outcomes. Expanding the scope of future analyses to include psychosocial variables would provide a more holistic understanding of how various factors contribute to disability among older adults.

### Recommendations

Based on the study’s findings and acknowledging its methodological limitations, several critical recommendations emerge to inform policy, programming, and future research on ageing and disability in Botswana. First, there is a pressing need to strengthen community-based and age-specific health services. As disability rates rise significantly with age, particularly after 75, integrating geriatric care into primary healthcare systems is essential. This includes improving access to early screening for functional impairments, expanding rehabilitative services such as physical therapy, and ensuring the availability of assistive devices across both urban and rural settings.

Second, the observed gender disparities in disability prevalence underscore the importance of promoting gender-sensitive ageing policies. Older women, especially widows and those with limited employment histories, require targeted social protection measures. These may include cash transfers, tailored health services, and strengthened community support structures that acknowledge and mitigate the cumulative disadvantages women face over the life course.

Education also emerged as a powerful protective factor, highlighting the value of lifelong learning. National strategies should thus invest in adult education and health literacy initiatives that empower older persons to effectively manage chronic conditions, make informed health decisions, and navigate the healthcare system. In tandem, enabling older adults to remain economically and socially engaged is crucial. The association between employment and reduced disability suggests the need for age-friendly labour policies, flexible work opportunities, and community engagement programs that reduce isolation and promote active ageing.

Improving the living conditions of older adults must also be prioritized. The study identified small and overcrowded households as risk factors for disability, indicating the need for affordable, accessible, and age-appropriate housing. Government housing strategies should support the development of new housing and subsidize retrofitting of existing homes to accommodate the mobility and care needs of older residents.

Moreover, addressing the urban–rural divide in disability outcomes demands equitable investment in infrastructure. Older adults in rural areas face higher risks due to limited access to healthcare, transport, and technology. Investment in health posts, transportation systems, road networks, and digital connectivity in rural areas is vital to enable healthy ageing in place and to close spatial inequities.

Digital inclusion also plays a transformative role. Older adults in communities with internet, electricity, and computer access report significantly lower disability rates, highlighting the need for national ICT strategies that target older populations. These should include provision of affordable devices, digital literacy training, and development of age-friendly e-health platforms to support access to telemedicine and digital health resources.

Transportation access was found to be another important determinant. Policymakers must prioritize the development of inclusive transport systems, including age-friendly public transit and community transport options that enable older persons to maintain their autonomy, reach healthcare services, and remain socially connected.

Additionally, while the study focused primarily on structural and environmental factors, it is essential to incorporate psychosocial dimensions such as mental health, loneliness, access to mental health services, social connectedness, perceptions of ageing and caregiver support into future research and policy design. These factors are central to the lived experience of disability and should be integrated into holistic interventions.

Lastly, the cross-sectional nature of the data limited causal inference. Therefore, longitudinal research is imperative to capture the progression and determinants of disability over time. Such studies would offer deeper insights into the dynamics of functional decline and provide an empirical foundation for the design of proactive, responsive, and sustainable ageing policies in Botswana.

## Supplementary Information

Supplementary Material 1

## Data Availability

The data supporting the findings of this study are accessible through Statistics Botswana and can be obtained by researchers upon request. Additionally, the data may be provided by the authors upon reasonable request, subject to approval from Statistics Botswana.

## Abbreviations

ADLs: Activities of Daily Living
AIC: Akaike’s Information Criterion
AICC: Finite Sample Corrected Akaike’s Information Criterion
BIC: Bayesian Information Criterion
CAIC: Consistent Akaike’s Information Criterion
CI: Confidence Interval
GLM: Generalized Linear Model
ICF: International Classification of Functioning, Disability and Health
ICT: Information and Communication Technology
IRB: Institutional Review Board
IRR: Incidence Rate Ratio
LMICs: Low- and Middle-Income Countries
NB: Negative Binomial
PHC: Population and Housing Census
SPSS: Statistical Package for the Social Sciences
UB: University of Botswana
UN DESA: United Nations Department of Economic and Social Affairs
WGSS: Washington Group Short Set
WHO: World Health Organization
