# Supplementary material for: Determinants of old age disability in Botswana: an empirical investigation using generalized linear models
Source: BMC Geriatr. 2025 Nov 7;25:864. doi: 10.1186/s12877-025-06534-z (PMC12595874; doi:10.1186/s12877-025-06534-z)
Supplement: Supplementary file 1 — Supplementary Material 1 [file 12877_2025_6534_MOESM1_ESM.pdf]

Policy Brief: Determinants of Old-Age Disability in Botswana

Author: Theodore Monamo

Background

Botswana is undergoing a demographic transition, with adults aged 65+ now representing 5.5% of the population, a proportion expected to rise significantly. With ageing comes an increased burden of disability, affecting independence, quality of life, and health system demand. Disability among older adults is not only the result of biological ageing but is shaped by intersecting individual, household, and community-level factors. Understanding these determinants is critical for designing inclusive, multisectoral ageing policies.

Study Overview

This study analysed data from 47,309 older adults (65+) drawn from the 2022 Botswana Population and Housing Census, using Generalized Linear Models (Poisson regression). Disability was measured across three functional domains: mobility, self-care, and cognition. The analysis examined the role of demographic, socioeconomic, household, and community-level determinants of disability.

Key Findings

- **Age and Gender:** Disability prevalence increases markedly after age 75. Women are disproportionately affected, reflecting cumulative social and economic disadvantages.
- **Education and Employment:** Older adults with primary or less education are 20% more likely to experience disability. Employment is protective, with employed older adults 30% less likely to be disabled.
- **Marital Status:** Being married or never married is associated with lower disability compared to being widowed, divorced, or separated.
- **Household Context:** Living in smaller or overcrowded households (1–2 rooms) increases disability risk by up to 9%.
- **Community Infrastructure:** Access to electricity, internet, computers, and transport (private, public, or non-motorized) is associated with lower disability rates. Rural residence significantly increases disability risk compared to urban and urban village settings.

Policy Implications

1. **Strengthen Age-Sensitive Health Services:** Integrate geriatric care into primary health systems, expand rehabilitation, and ensure access to assistive devices.
2. **Promote Gender-Sensitive Interventions:** Address women's cumulative disadvantages with tailored social protection, caregiver support, and equitable access to health services.
3. **Invest in Lifelong Learning:** Promote adult education and health literacy programs to reduce long-term disability risks.
4. **Support Active Ageing:** Develop age-friendly labour policies to extend economic and social engagement opportunities for older adults.
5. **Improve Living Environments:** Expand affordable, age-appropriate housing and retrofitting programs to address risks from overcrowding and poor infrastructure.
6. **Reduce Urban–Rural Inequities:** Prioritize investment in rural infrastructure (transport, health posts, electricity, and digital connectivity) to close disability gaps.
7. **Advance Digital Inclusion:** Implement ICT strategies that enhance older adults' digital literacy and access to affordable devices, enabling telehealth and online services.
8. **Ensure Mobility:** Design inclusive transportation systems that facilitate healthcare access, independence, and social participation.

Conclusion

Old-age disability in Botswana is shaped by demographic, socioeconomic, and infrastructural determinants. Addressing these requires integrated, cross-sectoral action that goes beyond healthcare to include housing, transport, digital inclusion, and gender equity. As Botswana's older population grows, proactive investment in age-friendly, inclusive policies will be essential to promote independence, dignity, and wellbeing among older citizens.

Audience: Policymakers in health, social protection, housing, ICT, and transport; civil society; development partners.

Keywords: Ageing, Disability, Botswana, Health Policy, Infrastructure, Digital Inclusion, Active Ageing
